# Supplementary material for: Hawler Medical University students’ perceptions of e-learning during the COVID-19 pandemic
Source: PLoS One. 2023 Feb 23;18(2):e0281117. doi: 10.1371/journal.pone.0281117 (PMC9949649; doi:10.1371/journal.pone.0281117)
Supplement: Attachment — Submitted filename: Response_to_Reviewers.docx [file pone.0281117.s003.docx]

**PLOS One**

**Re: Submission of the revised manuscript**

Dear Editor,

Thank you very much for sharing the Editor's and reviewers' comments and suggestions on my manuscript entitled " Hawler Medical University students’ perceptions of e-learning during the COVID-19 pandemic." I very much thank them for their valuable comments and suggestions. These comments have significantly helped me to improve the quality and clarity of the manuscript.

I have made the necessary revision by responding to the suggested comments. Please find below explanations to the revision made through a point-to-point response to the comments.

Thank you very much for considering the revised manuscript.

Best regards,

Sherzad Shabu

**Journal Requirements:**

1. Please ensure that your manuscript meets PLOS ONE's style requirements, including those for file naming.

**Author's response**

PLOS ONE style requirements are applied to the manuscript.

2. Please provide additional details regarding participant consent. In the ethics statement in the Methods and online submission information, please ensure that you have specified what type you obtained (for instance, written or verbal, and if verbal, how it was documented and witnessed). If your study included minors, state whether you obtained consent from parents or guardians. If the need for consent was waived by the ethics committee, please include this information

**Author's response**

Online written consent was obtained from the participants before completing the survey.

Please review your reference list to ensure that it is complete and correct. If you have cited papers that have been retracted, please include the rationale for doing so in the manuscript text, or remove these references and replace them with relevant current references. Any changes to the reference list should be mentioned in the rebuttal letter that accompanies your revised manuscript. If you need to cite a retracted article, indicate the article’s retracted status in the References list and also include a citation and full reference for the retraction notice.

**Author's response**

It was checked and it is complete and correct.

**Additional Editor comment**

Please revise the manuscript. It has been mentioned in the methodology that "The questionnaire was 117 developed based on reviewing the literature of similar studies." Therefore, please provide the references for the questionnaire.

**Author's response**

The reference for the questionnaire was provided (Reference number 9). The statement was also revised under the Methods section/Survey tool, Page 6, Line number 117 and 118.

**Review Comments to the Author**

**Reviewer #1:**

**Reviewer’s comment**

Good effort on a burning issue. The flow of writing is fine and ideas are presented in a well structured manner. Problem and knowledge gap are identified. Results are discussed well but they are not compare enough with other studies and not critically analyze enough. I suggest if the provide further recommendations based on the conclusion of study.

**Author's response**

Thank you very much for the valuable comments.

- Further comparisons of results were made under the Discussion section (Page 19, Line 277-284) and (Page 20-21, Line 314-323).

- Critical analysis of results was made under the Discussion section (Page 18, Line 254-256), (Page 19, Line 288-290) and (Page 21, Line 325-327).

- Further recommendations were provided based on the conclusion of the study under the Conclusions section (Page 22, Line 363-370).

**Reviewer #2:**

**Reviewer’s comment**

However, in the methodology section the development of the tool especially with respect to the selection of the items needs more emphasis. The section says that the items were taken from literature from similar studies, but it is not clear whether the items were adapted from any similar survey questionnaire. Furthermore, whether the tool was piloted has not been mentioned. In the analysis it would have been beneficial if the authors would have considered the comparison between different participant groups.

**Author's response**

Thank you very much for the useful comments.

- The questionnaire was adapted from a study conducted to develop a questionnaire for predicting online learning achievement (Reference number 9). The statement was added to the Methods section/Survey tool (Page 6, Line 117-118).

- Piloting the survey tool: A paragraph was added in Methods section/Survey tool (Page 7, Line 141-145) as follows:

For each stage, and prior to data collection, ten Hawler Medical University students were initially invited to pilot test the questionnaire. They completed the online survey questionnaire and tested the clarity of the questions' content and the practicality of the questionnaire's completion. Based on the comments from these students, the questionnaire was adapted.

- Comparison between different participant groups in the analysis: A "Study limitations" section was added at the end of the Discussion section (Page 22, Line 350-353) stating the following:

# Study limitations

Because of the study design and mainly using the Likert scale for data collection and organization, it was somehow difficult to consider comparison between different participants' groups. Further studies which consider comparison between these groups are recommended.
